# An Infant With Congenital Tracheal and Bronchial Stenosis Diagnosed by Chest Three-Dimensional Computed Tomography

**DOI:** 10.7759/cureus.24771

**Published:** 2022-05-06

**Authors:** Yuko Moriuchi, Tatsuo Fuchigami, Waka Mizukoshi, Ichiro Morioka

**Affiliations:** 1 Department of Pediatrics, IMS Fujimi General Hospital, Saitama, JPN; 2 Department of Pediatrics and Child Health, Nihon University School of Medicine, Tokyo, JPN; 3 Department of Radiology, IMS Fujimi General Hospital, Saitama, JPN

**Keywords:** virtual bronchoscopy, chest 3d-ct, recurrent airway infections, prolonged wheezing, congenital tracheal stenosis

## Abstract

In this case report, we describe the case of an infant with repeated wheezing diagnosed relatively early with congenital tracheal and bronchial stenosis after evaluation by chest three-dimensional computed tomography (3D-CT). The patient was a six-month-old male infant with a one-month history of cough and wheezing. His symptoms worsened the day before admission, and he was admitted with pneumonia and wheezing. However, wheezing continued after treatment with intravenous steroids and inhalation of a short-acting β2-stimulant. 3D-CT of the chest revealed tracheal stenosis, right bronchial stenosis, and right tracheobronchial bronchus.

The patient was finally diagnosed with congenital tracheal and bronchial stenosis via bronchoscopy. A virtual bronchoscopic navigation image of the tracheal lumen was created based on the CT images. Although virtual bronchoscopic navigation is more difficult for the dynamic evaluation and evaluation of mucosal lesions than bronchoscopy, it has the advantage of not directly invading the airway. Therefore, if a fixed stenotic lesion is suspected at a facility where bronchoscopy is difficult, evaluation using chest 3D-CT and virtual bronchoscopic navigation may be helpful for diagnosis. In conclusion, congenital tracheal/bronchial stenosis should be considered in patients with prolonged wheezing and recurrent airway infections, and evaluation by chest 3D-CT and virtual bronchoscopic navigation may be helpful for diagnosis.

## Introduction

Congenital tracheal stenosis is a disease that causes narrowing of the airway lumen due to the abnormal formation of complete tracheal cartilage rings without tracheal membrane-like portions and is estimated to occur in one case per 65,000 live births [1]. The onset varies depending on the range and extent of stenosis, but many cases present as wheezing within a few months of age, often with respiratory infections, which causes rapid worsening of symptoms [1]. However, some cases are regarded as atypical "respiratory distress" and atypical "croup" or "bronchiolitis," and diagnosing congenital stenosis takes time [2].

In this case report, we describe the case of an infant with repeated wheezing diagnosed relatively early with congenital tracheal and bronchial stenosis after evaluation by three-dimensional computed tomography (3D-CT) of the chest.

## Case presentation

The patient was a six-month-old male infant. Prolonged wheezing and coughing started at the age of five months, for which he was treated at another hospital. Wheezing and cough worsened the day before admission, and nasal discharge was observed. Hyperpnea, retractive breathing, and oxygen demand were observed when the patient visited another hospital on the day of admission. The patient was subsequently transferred to our hospital. He was delivered at a gestational age of 40 weeks and three days (birth weight: 3,425 g; Apgar score: 8/9) by emergency cesarean section due to decreased fetal heart rate. Surgical treatment for intestinal malrotation was performed at five days of age at another hospital, and the patient was also being observed for patent ductus arteriosus at another hospital. His growth and developmental history were average, including a head control at three months and turning over at six months. The patient’s family history was unremarkable. His vaccination history stated that he was inoculated with age-appropriate vaccines, and his life history stated that he has used group childcare and has no food or drug allergies.

On admission, his height was 63 cm (-2.0 SD), and his weight was 7.5 kg (-0.56 SD). His Kaup index was 18.9; body temperature was 37.5°C; heart rate was 175 beats/min; the respiratory rate was 40 breaths/min; oxygen saturation (room air) was 90%, and poor mood, no pale face, no pharyngeal redness, no thoracic deformity, and wheezing during inspiration and expiration in bilateral lungs were observed. No rash or atopic dermatitis. 

The results of the laboratory findings on admission are as follows: Blood tests revealed a mildly elevated white blood cell count of 10,500/µL and CRP of 0.34 mg/dL. Venous blood gas analysis showed no acidosis or CO_2_ retention, and the rapid respiratory syncytial virus antigen test was negative. Postnasal culture revealed *Branhamella catarrhalis* (2+) and penicillin-sensitive *Streptococcus pneumonia* (1+). A chest radiograph showed an infiltrative shadow in the right S6 region.

After admission, the patient was diagnosed with pneumonia with wheezing and was started on intravenous methylprednisolone 2 mg/kg/day, inhaled short-acting beta-stimulant, expectorant, and bronchodilator medication, and oxygen administration. Although wheezing was heard, oxygen administration was no longer required from the fifth day of hospitalization. On the seventh day of hospitalization, the patient was discharged from the hospital as the chest radiograph showed improvement in the shadow of pneumonia. However, wheezing persisted even after discharge; therefore, the patient was treated with oral leukotriene receptor antagonists and inhalation of budesonide and short-acting beta-stimulants in consideration of infantile asthma. Additional treatment with oral dexamethasone and antibacterial agents was administered, but all were refractory to treatment.

**Figure 1:**
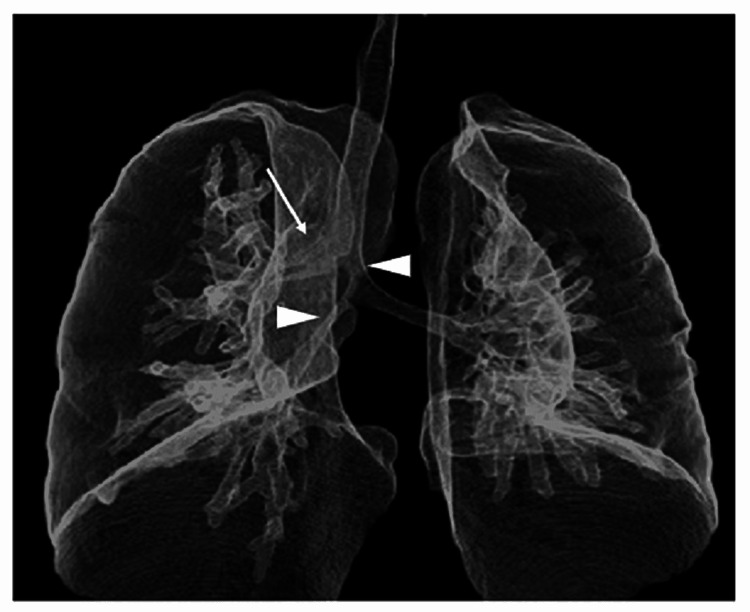
3D-CT of the chest ▷ indicates stenosis, and → indicates tracheobronchus. 3D-CT: Three-dimensional computed tomography.

A chest radiograph at seven months showed a right pneumonia shadow; therefore, detailed examinations for the organic disease were performed. Blood tests showed no increase in pertussis antibody titer. The IgE radioallergosorbent test (RAST) showed no increase in specific IgE antibodies for house dust, mites, cow's milk, beta-lactoglobulin, or casein. Next, considering congenital airway lesions, a simple CT of the chest was performed, which revealed infiltrative shadows in the right middle and lower lobes; 3D-CT construction revealed tracheal stenosis, right tracheobronchial stenosis, and right bronchial stenosis (Figure 1), with no vascular malformations such as vascular rings or pulmonary artery slings.

**Figure 2:**
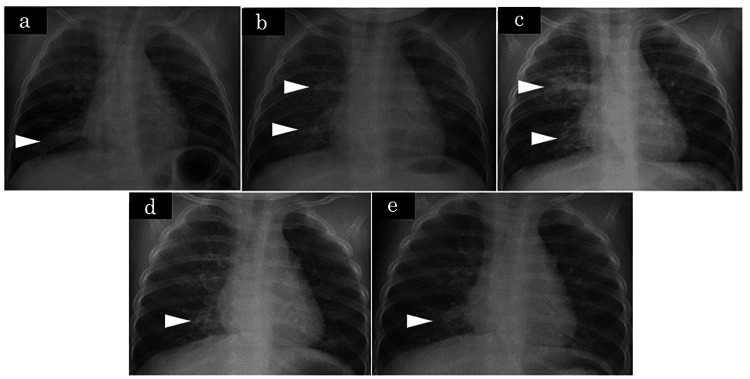
Chest XP Chest XP at (a) Month 6, (b) Month 7, (c) Month 8, after bronchoscopy, (d) Month 10, and (e) Month 11. ▷ shows an image of pneumonia. XP: X-ray photography.

As it was difficult to evaluate the patient by bronchoscopy at our hospital, we referred him to the Department of Respiratory Medicine at another hospital. He was diagnosed with congenital tracheal and bronchial stenosis by bronchoscopy. Conservative treatment was chosen because the diameter of the trachea and bronchus at the stenosis area was not narrow, and the area was limited. Although wheezing was recognized, there was no respiratory disturbance except during airway infection. At the respiratory department of another hospital, the patient was treated with daily oral expectorant medication and sodium cromoglycate inhalation. At our hospital, the policy is to deal with respiratory tract infections while paying attention to the deterioration of the respiratory condition. Subsequently, right-sided pneumonia was observed five times by the age of one year (Figure 2). Still, it was not observed after the age of one year, and wheezing became less noticeable, so conservative treatment was completed. The patient is now three years of age and is doing well.

## Discussion

**Figure 3:**
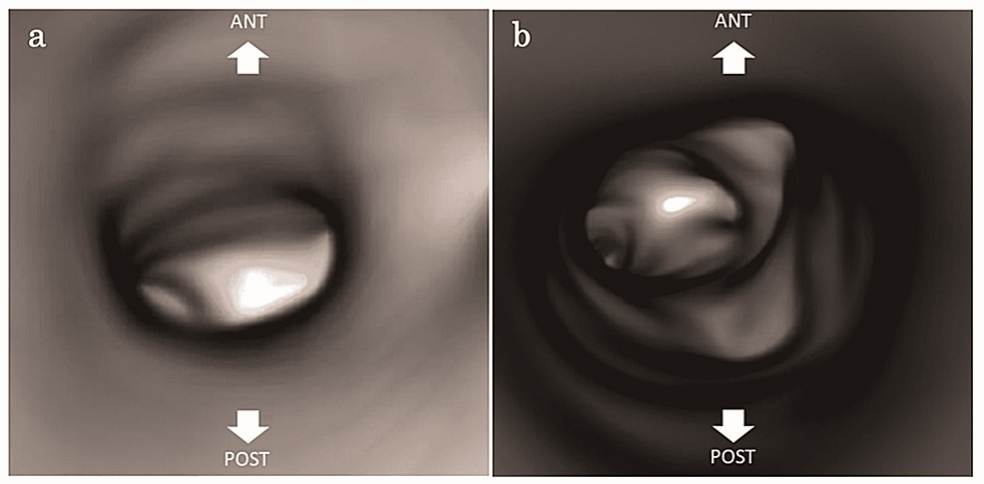
Virtual bronchoscopic navigation (a) Tracheal stenosis. (b) Right bronchial stenosis.

Bronchoscopy is necessary for diagnosing congenital tracheal stenosis and acquired cases such as postintubation tracheal stenosis. Still, in recent years, reports have suggested that 3D-CT of the chest and virtual bronchoscopic navigation are also useful [3-8]. By reviewing the initial chest radiograph in this case (Figure 2, Panel a), it was possible to suspect a congenital airway lesion based on the chest x-ray findings as we noted that the trachea was more deviated to the right than usual when it branched to the right and that the position of the bifurcation was higher. However, findings of tracheal stenosis, right bronchial stenosis, and tracheobronchial tubes were more evident on chest 3D-CT. Furthermore, when the tracheal lumen images were created by virtual bronchoscopic navigation based on the CT images, the findings of tracheal stenosis were unclear; however, the right bronchial stenosis appeared to be a complete tracheal cartilage ring (Figure 3).

Bronchoscopy has several advantages, including the ability to obtain dynamic images and confirm mucosal lesions by color tone; however, it also has disadvantages such as the need for general anesthesia and varying accuracy depending on the skill level of the physician [7]. Conversely, virtual bronchoscopic navigation has the advantage that it can be performed without anesthesia, and there is no direct invasion of the airway. However, dynamic evaluation and evaluation of the mucosal lesions are difficult [7]. In this case, right-sided pneumonia was observed after bronchoscopy, suggesting that airway stenosis was aggravated by the invasion caused by bronchoscopy, resulting in pneumonia. In light of this, if a fixed stenotic lesion is suspected at a facility where bronchoscopy is difficult, evaluation by chest 3D-CT and virtual bronchoscopy navigation should be performed first. If the stenotic lesion is unclear or surgical treatment is required, it may be helpful to evaluate the origin and extent of the stenosis under direct visualization by bronchoscopy. Therefore, it should be referred to a facility that can treat bronchoscopy as soon as possible.

In congenital tracheal stenosis, when the degree of airway stenosis and respiratory symptoms are mild, the stenotic area expands as the child grows, and the symptoms decrease [9-11]. In this case, conservative treatment was chosen because the diameter of the bronchus was approximately 2-3 mm, the area was localized, and the respiratory symptoms abated with growth. However, infants are prone to airway stenosis because of their relatively small airway diameter and soft airway smooth muscles. Therefore, it is necessary to remember that respiratory distress progresses rapidly during airway infection and may lead to asphyxia in infants with congenital airway stenosis.

## Conclusions

Here, we reported the case of an infant with repeated wheezing who was diagnosed relatively early with congenital tracheal and bronchial stenosis after evaluation using 3D-CT of the chest. Congenital tracheal and bronchial stenosis should be considered in prolonged wheezing and recurrent airway infections, and evaluation using chest 3D-CT and virtual bronchoscopic navigation may be helpful for diagnosis.
